# Sterile inflammation – do innate lymphoid cell subsets play a role?

**DOI:** 10.3389/fimmu.2012.00246

**Published:** 2012-08-07

**Authors:** Shane E. Russell, Patrick T. Walsh

**Affiliations:** ^1^Department of Clinical Medicine, School of Medicine, Trinity College Dublin,Dublin, Ireland; ^2^National Children’s Research Centre, Our Lady’s Children’s Hospital, Crumlin,Dublin, Ireland

**Keywords:** sterile inflammation, innate lymphoid cell, γδ T cell, type 1 response, type 2 response

## Abstract

The recent identification of several novel innate lymphoid cell (iLC) subsets has increased our understanding of the mechanisms which link the innate and adaptive immune systems. While the contribution of these subsets toward the pathogenesis of human disease remains largely to be determined, it seems likely that they will play a particularly important role in sterile inflammatory settings where the innate response is seen as a critical mediator of inflammation. Several recent studies have highlighted the role of endogenous damage-associated molecular patterns such as IL-33, IL-1α, and IL-1β in promoting lymphoid cell responses. This review discusses the influence of such endogenous danger signals on novel iLCs such as lymphoid tissue-inducer cells, innate type 2 helper cells, and γδ T cells and explores how these responses may contribute to the development of an inflammatory response in a sterile setting.

## INTRODUCTION

Acute inflammation directed by the innate immune response has evolved to efficiently combat infection and is critical to host defense. However, in the absence of overt infection, such innate mechanisms may also be activated as a result of tissue injury resulting from metabolic or exogenous sources (Rock et al., 2010). These events lead to what is termed a sterile inflammatory response which, if not appropriately resolved, can lead to the development of chronic inflammation which underlies or exacerbates a variety of human diseases.

The driving signals of sterile inflammation are known as damage-associated molecular patterns (DAMPs) and while distinct from their pathogen-derived counterparts, they elicit their effects through many of the same signaling pathways and networks (Bianchi, 2007). As a result, much of what we know about the mechanisms involved in sterile inflammation has stemmed from investigations of the innate inflammatory response to infection. Indeed, as a result of collateral damage often observed during the innate response to infection, DAMPs can also contribute to inflammation in non-sterile settings (Yang and Oppenheim, 2009; Yang et al., 2009; Andersson and Tracey, 2011). Most studies on the sterile inflammatory response have focused on the sources of these endogenous DAMPs, such as necrotic cells (Krysko et al., 2011). However, there is a growing awareness of the influence DAMPs play in orchestrating sterile inflammation through activating different cell types (Nace et al., 2012). This review will focus on the effects of specific DAMP activity on recently defined innate lymphoid cell (iLC) populations and how these distinct populations of cells play a role in orchestrating subsequent inflammatory events relevant to human disease.

## DAMAGE-ASSOCIATED MOLECULAR PATTERNS

Damage-associated molecular patterns (DAMPs) are proinflammatory mediators largely activated under conditions of cellular stress or injury which may ultimately result in cell death. Stimuli which elicit these responses can range from sterile particulates derived from either aberrant metabolism, e.g., cholesterol crystals in atherosclerosis (Duewell et al., 2010) or external toxins, e.g., asbestos (Dostert et al., 2008). Similarly, unprogramed cell death, such as that observed under ischemic conditions or chemotherapeutic treatment strategies, leads to a loss of cellular integrity which can result in the exposure of DAMPs and the development of sterile inflammation (Arslan et al., 2011; Awong et al., 2012). In recent times, the identity of several DAMPs has been revealed and their effects on the generation of the sterile immune response described. As this review will focus on how these signals specifically influence iLCs responses, a number of the most relevant DAMPs are described below.

## IL-1 α/β

The proinflammatory effects of both IL-1α and IL-1β have long been recognized. Although both DAMPs share the same IL-1 receptor, and thus appear to elicit very similar effects, their expression and activity are distinctly regulated (Dinarello, 2009). IL-1β gene expression is induced upon stimulation by numerous inflammatory signals but is normally translated in its inactive proform. Prior to its release from cells IL-1β undergoes maturation through proteolytic cleavage by caspase-1 in events mediated by an inflammasome involving the proteins NLRP3 and ASC (Li et al., 1995; Lamkanfi and Dixit, 2009). Critically, activation of the signaling events required for the appropriate assembly and activation of this NLRP3 inflammasome are known to be mediated by a wide range of sterile particulates and metabolites implicating IL-1β as central to numerous sterile inflammatory conditions (Martinon et al., 2006; Dostert et al., 2008; Masters et al., 2010). Unlike IL-1β, IL-1α appears to be constitutively expressed by a wider range of cell types and although it also undergoes proteolytic cleavage, is active in its unprocessed form. Although IL-1α does not appear to require inflammasome activity for its activation it is known to be released in large amounts upon cell death through necrosis and has been found play a central role in driving neutrophil recruitment as part of the sterile inflammatory response (Chen et al., 2007).

The effects of IL-1 activity on both the innate and adaptive immune responses have been extensively characterized (Dinarello, 2009), and there is accumulating evidence that these DAMPs play an important role in driving iLC subset responses. These events are likely to play a significant role in driving neutrophil recruitment under sterile conditions and are discussed in greater detail below.

## IL-33

IL-33 is another member of the IL-1 family of cytokines which is exposed upon cellular injury and acts upon its cognate receptor ST2 in complex with the IL-1RAcP (Schmitz et al., 2005). Although originally thought to be post transcriptionally regulated by caspase-1-mediated cleavage, in a manner similar to IL-1β, it is now apparent that IL-33 exhibits biological activity in its full-length uncleaved form and is released upon necrotic cell death to exert its biological activity in a similar manner to IL-1α (Liew et al., 2010). As well as acting as a classical “alarmin,” intracellular IL-33 can also act as a transcriptional repressor although the specific genes and pathways which it targets in this role are not well-defined (Carriere et al., 2007; Ali et al., 2011). Release of IL-33 is strongly implicated as an instructive signal in the development of type 2 immune responses and as such it is thought to play an important role in the allergic response (Schmitz et al., 2005). More recently it has been demonstrated that IL-33 has profound effects on innate type 2 helper cells and nuocytes and may play a central role in driving type 2 immunity under sterile settings (Kim et al., 2012; Mirchandani et al., 2012). The influence of IL-33 on iLC populations in the context of sterile inflammation is discussed in further detail below.

## EFFECTS OF DAMPs ON INNATE LYMPHOID CELL SUBSETS

The effects of DAMP signaling on cells of the innate immune system such as dendritic cells and macrophages have been extensively described elsewhere (Chen and Nunez, 2010). Similarly, since their earliest identification there have been numerous studies detailing how endogenous DAMPs can directly influence the adaptive T cell response. For example, HMGB1, CpG containing DNA motifs, and hsp60 have all been described to directly influence activated T cell responses (Dumitriu et al., 2005; Zanin-Zhorov et al., 2006; LaRosa et al., 2007). In more recent times there has been an intense interest in identifying novel lymphoid cell subsets which are thought to play an important role in bridging the gap between the innate and adaptive immune responses. These subsets are largely segregated by their ability to rapidly express effector cytokines more commonly associated with adaptive T helper cell responses such as IL-17 related cytokines or IL-13 and IL-4. Perhaps unsurprisingly, this has led to significant advances in understanding the role of these subsets in both autoimmune and allergic disease settings (Cai et al., 2011; Barlow et al., 2012; Pantelyushin et al., 2012). However there is significant evidence to suggest that such subsets may also play a central role in inflammation under sterile settings. In particular, the observation that proinflammatory cytokines normally associated with adaptive T cell responses are elevated in certain auto-inflammatory disease settings, in the absence of any identified sources of antigenic stimulation, indicates that iLCs may play an important role (Lasiglie et al., 2011).

## INNATE LYMPHOID CELL SUBSETS IMPLICATED IN STERILE INFLAMMATION

### γδ T CELLS

Arguably the most extensively described iLC populations are γδ T cells, which comprise about 5% of the overall T cell population. They differ from conventional αβ T cells in that they express invariant γ and δ chains as part of their T cell receptor and are resident predominantly at mucosal sites (Cua and Tato, 2010). γδ T cells appear to lack the requirement for conventional antigen presentation and this has contributed to the hypothesis that these cells act as tissue-resident immune sentinel cells. Of particular relevance to sterile inflammatory conditions are the recent observations from both mouse and human studies that specific subsets of γδ T cells respond to IL-R1 stimulation by expressing significant levels of IL-17A (Sutton et al., 2009; Ness-Schwickerath et al., 2010; Caccamo et al., 2011). These cells which constitutively express CCR6 and the transcription factor RORγT may act as an important instructive signal to promote the generation of adaptive Th17 type responses but have also been implicated as important mediators of disease in their own right (Sutton et al., 2009). In particular, γδ T cells which express IL-17A in response to IL-1α/β play an important role in driving neutrophilia and dermal inflammation in psoriasis in both mouse models and human patients (Cai et al., 2011; Laggner et al., 2011; Pantelyushin et al., 2012). Specifically, human Vγ9Vδ2^+^ cells, which are induced to express IL-17A in the presence of IL-1β, have been implicated as playing a prominent role in psoriasis (Laggner et al., 2011). As neutrophilic skin inflammation is a prominent feature of cryopyrin-associated inflammatory syndrome (CAPS), which are autoinflammatory disorders, associated with inflammasome hyperactivity and increased IL-1β processing, it is tempting to speculate that Vγ9Vδ2^+^ T cells may play a role in mediating these events (Kolivras et al., 2011). Indeed, in a recent study, significantly elevated levels of IL-17A was found in the serum of CAPS patients and although CD4^+^ Th17 cells were implicated as the source of IL-17A, the status of γδ T cell subsets was not examined (Lasiglie et al., 2011). Similarly, characterizations of Nlrp3 gene-targeted mice harboring mutations which mimic those causing disease in humans have demonstrated significant skewing toward a Th17 type response. Of particular interest in this regard, Meng et al. (2009) have demonstrated that mice expressing a R258W mutation (corresponding to the R260W mutation found in Muckle–Wells disease patients) exhibit spontaneous skin inflammation associated with neutrophil inflammation and a Th17 dominated response. As this phenotype bears a remarkable similarity to psoriasis, in which γδ T cells are a prominent source of IL-17 related cytokines, it raises the possibility that γδ T cells may also play an important role in skin manifestations among CAPS patients.

**FIGURE 1:**
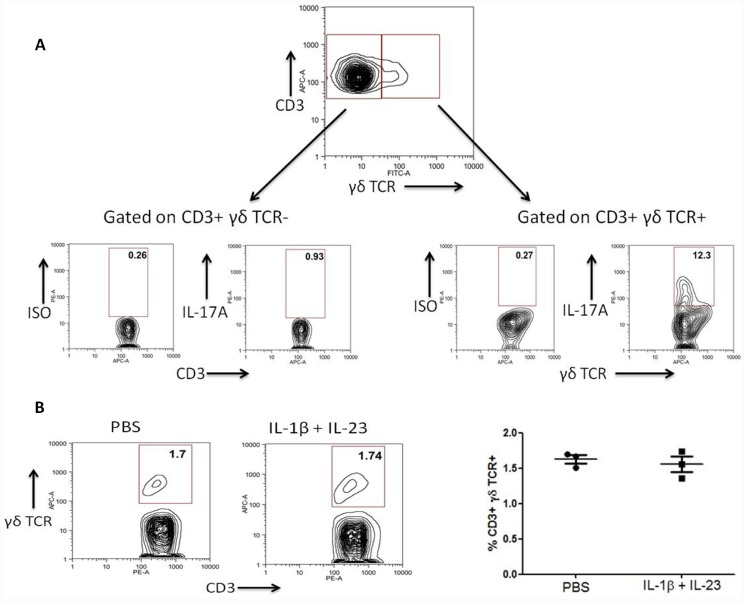
**IL-1β and IL-23 induce rapid expression of IL-17A by γδ T cells *in vivo***. C57Bl/6 mice were injected into the hind footpad with IL-1β (50 ng) and IL-23 (50 ng) or PBS. After 4 h cells were harvested from the popliteal lymph nodes and treated for a further 4 h with brefeldin A without *ex vivo* stimulation. Cells were stained for expression of CD3, γδ TCR and intracellular IL-17A or isotype control and examined by flow cytometry. **(A)** Data shown as IL-17A expression by live CD3^+^γδTCR^–^ and CD3^+^γδTCR^+^ cells is representative of four separate experiments. **(B)** The percentage frequency of CD3^+^γδTCR^+^ cells is unaltered after injection with IL-1β and IL-23. Data representative of three individual mice.

While it has been established that caspase-1-dependent IL-1β activity can promote expression of IL-17A by γδ T cells in autoimmune disease settings (Lalor et al., 2011), whether this occurs under sterile settings has not been investigated. We have found that the presence of IL-1β with IL-23 can induce significant rapid expression of IL-17A by murine γδ T cells *in vivo* under sterile settings and in the absence of exogenous antigen. In contrast, CD3^+^ γδ TCR^–^ cells do not express significant levels of IL-17A under these conditions (**Figure 1**). As both IL-1β and IL-23 have been shown to be expressed at higher levels by monocyte derived dendritic cells from CAPS patients (Lasiglie et al., 2011), this further indicates that γδ T cell subsets may provide an important innate source of IL-17 related cytokines in autoinflammatory disease.

## LYMPHOID TISSUE-INDUCER CELLS

Another prominent innate lymphoid subset characterized by their ability to quickly and efficiently express IL-17 and/or IL-22 are lymphoid tissue-inducer cells (LTi; Takatori et al., 2009). LTi cells require the activity of the RORγT transcription factor for their development and were initially identified for their role in driving lymphoid tissue development during embryogenesis. More recently, LTi have been found to play important roles in innate immune responses at mucosal surface such as the intestine and the skin where they have been implicated as playing an important role in responding to both pathogenic and commensal bacteria (Ivanov et al., 2006; Pantelyushin et al., 2012). However there is also emerging data indicating that these cells can play a prominent role in sterile inflammatory settings.

As well as the autoinflammatory syndromes referred to above, which result from genetic abnormalities, “sterile” inflammation can also occur as a result of therapeutic regimens designed to target specific cell populations for depletion. Treatments such as chemotherapy and irradiation often result in undesirable effects on the development of a healthy immune system with serious consequences for the patient. Recently, it has been demonstrated that LTi cells play a central role in promoting appropriate thymic regeneration in such sterile inflammatory settings (Dudakov et al., 2012). These effects are mediated largely through expression of IL-22 which promotes epithelial repair and tissue regeneration and highlight the role of iLCs in promoting the resolution of “sterile” inflammation (Dudakov et al., 2012). As an important source of IL-17A at mucosal sites, LTi cells have also been found play an important role in driving acute inflammatory responses to an array of both infectious and chemical stimuli (Ivanov et al., 2006; Pantelyushin et al., 2012). Although whether these cells can specifically respond to direct stimulation with DAMPs such as IL-1α/β (or indeed IL-33) has yet to be fully investigated, it is possible that they may also play an important role in bridging innate and adaptive immunity under sterile settings.

## INNATE TYPE-2 CELLS

The recent discovery of a number of subsets of innate immune cells expressing ST-2 and responding to IL-33 has advanced our understanding of this cytokine and further expanded its emerging role as an alarmin (Liew et al., 2010). One such cell type, the nuocyte, is an innate type 2 cell which was originally identified as an IL-25 responsive non-T/non-B, γ-common chain-dependent cell which provided IL-4, IL-5, and abundant amounts of IL-13 at the onset of helminth infection (Neill et al., 2010). Interestingly, nuocytes are also expanded upon treatment with IL-33 and cells treated in this manner also secreted IL-6, IL-10, and GM-CSF (Moro et al., 2010).

The ability of nuocytes to respond to IL-33 in this manner implicates a role for these cells in the immune response to cellular stress. It is established that tissue damage caused by factors including high free fatty acids and oxidative stress commonly leads to necrosis and release of IL-33 (Moussion et al., 2008; Cayrol and Girard, 2009). Whilst no direct role for nuocytes has been described under such conditions, it is tempting to speculate that these cells could potentially provide an important source for at least some of the cytokines expressed in these settings, including IL-5, IL-10, and most notably IL-13 (Miller et al., 2010). For example, in atherosclerotic lesions, where severe tissue damage and necrosis are involved, IL-33 is thought to play a protective role through its ability to switch what is a type 1 dominated response toward a less inflammatory type 2 response. IL-33 increases the levels of IL-4, IL-5, and IL-13 and reduces incidence of F4/80 positive macrophages and CD3 positive cells found in atherosclerotic lesions (Miller et al., 2008). These observations raise the interesting possibility that nuocytes could potentially provide a source of these cytokines and play a protective role in cardiac disease.

The natural helper (NH) cell is another recent addition to the expanding number of innate type 2 lymphoid cell populations (Koyasu and Moro, 2011). These cells are capable of producing large amounts of IL-5 and IL-13 when stimulated by IL-33 in the presence of IL-2, IL-7, or thymic stromal lymphopoietin (TSLP; Halim et al., 2012). Interestingly, and in contrast to the other described innate type 2 populations, NH cells are predominantly found in fat-associated lymphoid clusters where it is thought that they play important roles in maintenance of homeostasis in adipose tissues (Moro et al., 2010). Moreover, IL-33 and ST-2 expression in adipose tissue has been reported to play a protective role in obesity-driven sterile inflammation through promotion of a type 2 environment. These events were found to occur in association with switching of the macrophage response from a M1 to M2 phenotype raising the possibility that NH cells could also contribute to the regulation of the sterile inflammatory response to obesity by acting as an important orchestrator of the type 2 response (Miller, 2011).

Nuocytes and NH cells are two phenotypically similar cell subsets which respond to IL-33 and appear to play pathogenic roles in models of allergic and parasitic inflammation. However with the emerging importance of IL-33 as an endogenous danger signal further investigation into what role these subsets may play in mediating type 2 immunity under some settings of sterile inflammation could prove beneficial.

## CONCLUDING REMARKS

**FIGURE 2:**
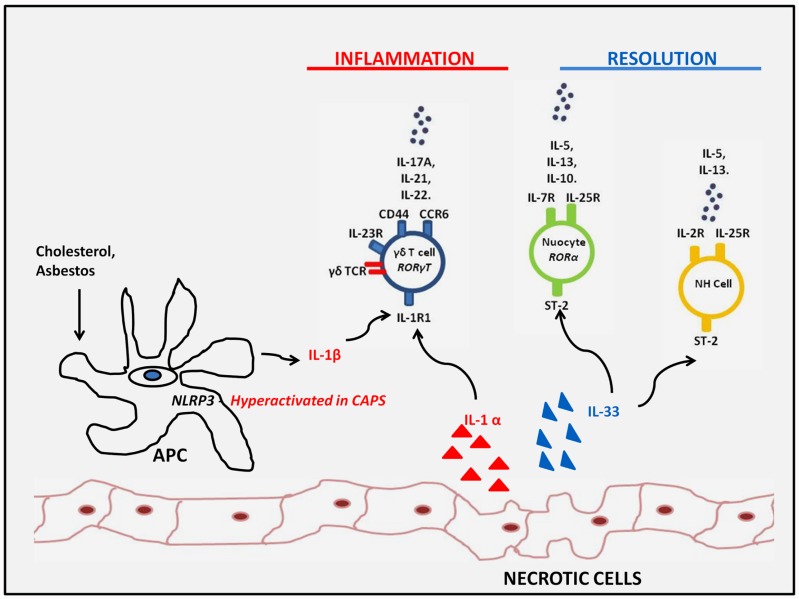
**Innate lymphoid subsets responding to sterile inflammation**. Innate lymphoid subsets such as γδ T cells and LTi cells may respond to increased IL-1β expression as a result of either genetic- or particulate-driven inflammasome activation or IL-1α exposed upon tissue damage. These DAMPs drive the innate expression of IL-17 family members which may contribute to sterile inflammation or IL-22 which can promote tissue regeneration. On the other hand, IL-33 exposed upon tissue damage, drives type 2 innate responses by nuocytes and NH cells which can have a proresolving influence and dampen sterile inflammatory responses observed in obesity and atherosclerotic plaques.

Although the innate immune response to DAMPs is critical to the initiation of sterile inflammation, this often occurs in association with enhanced expression of effector cytokines more commonly associated with adaptive T helper cell responses. The recent identification of several iLC subsets which can respond rapidly to innate stimuli by expressing such cytokines, in the absence of any obvious requirement for antigenic stimulation, indicates that these subsets may be important mediators of inflammation under sterile conditions. Interestingly, the studies highlighted above, demonstrate that these cells can play both proinflammatory and proresolving roles depending on the inflammatory setting and the effector cytokines which are expressed (**Figure 2**). Further investigation will likely reveal whether, as well as being critical regulators at the interface between innate and adaptive immunity under settings of infection and autoimmunity, these cells are also important mediators of sterile inflammation.

## Conflict of Interest Statement

The authors declare that the research was conducted in the absence of any commercial or financial relationships that could be construed as a potential conflict of interest.
